# GPR40 partial agonist MK-2305 lower fasting glucose in the Goto Kakizaki rat via suppression of endogenous glucose production

**DOI:** 10.1371/journal.pone.0176182

**Published:** 2017-05-23

**Authors:** Corin Miller, Michele J. Pachanski, Melissa E. Kirkland, Daniel T. Kosinski, Joel Mane, Michelle Bunzel, Jin Cao, Sarah Souza, Brande Thomas-Fowlkes, Jerry Di Salvo, Adam B. Weinglass, Xiaoyan Li, Robert W. Myers, Kevin Knagge, Paul E. Carrington, William K. Hagmann, Maria E. Trujillo

**Affiliations:** 1 Departments of Translational Imaging Biomarkers, Merck & Co., Inc., Kenilworth, New Jersey, United States of America; 2 In Vivo Pharmacology, Merck & Co., Inc., Kenilworth, New Jersey, United States of America; 3 In Vitro Pharmacology, Merck & Co., Inc., Kenilworth, New Jersey, United States of America; 4 Cardio-Metabolic Diseases, Merck & Co., Inc., Kenilworth, New Jersey, United States of America; 5 David H Murdock Research Institute, Kannapolis, North Carolina, United States of America; 6 Chemistry, Merck & Co., Inc., Kenilworth, New Jersey, United States of America; CRCHUM-Montreal Diabetes Research Center, CANADA

## Abstract

GPR40 (FFA1) is a fatty acid receptor whose activation results in potent glucose lowering and insulinotropic effects *in vivo*. Several reports illustrate that GPR40 agonists exert glucose lowering in diabetic humans. To assess the mechanisms by which GPR40 partial agonists improve glucose homeostasis, we evaluated the effects of MK-2305, a potent and selective partial GPR40 agonist, in diabetic Goto Kakizaki rats. MK-2305 decreased fasting glucose after acute and chronic treatment. MK-2305-mediated changes in glucose were coupled with increases in plasma insulin during hyperglycemia and glucose challenges but not during fasting, when glucose was normalized. To determine the mechanism(s) mediating these changes in glucose metabolism, we measured the absolute contribution of precursors to glucose production in the presence or absence of MK-2305. MK-2305 treatment resulted in decreased endogenous glucose production (EGP) driven primarily through changes in gluconeogenesis from substrates entering at the TCA cycle. The decrease in EGP was not likely due to a direct effect on the liver, as isolated perfused liver studies showed no effect of MK-2305 *ex vivo* and GPR40 is not expressed in the liver. Taken together, our results suggest MK-2305 treatment increases glucose stimulated insulin secretion (GSIS), resulting in changes to hepatic substrate handling that improve glucose homeostasis in the diabetic state. Importantly, these data extend our understanding of the underlying mechanisms by which GPR40 partial agonists reduce hyperglycemia.

## Introduction

GPR40 is predominantly expressed on the pancreatic β-cell and augments glucose stimulated insulin secretion (GSIS) *in vitro* and *in vivo* [1–6]. GPR40 agonist-mediated GSIS effects *in vivo* have been associated with glycemic improvements in lean, insulin resistant, and diabetic rodents [2,5,7,8]. Fasiglifam (TAK-875), a GPR40 partial agonist evaluated in clinical trials, demonstrated significant reductions in oral glucose tolerance, fasted plasma glucose, and hemoglobin HbA_1C_ [9,10]. However, the reductions in glucose during fasting as well as glucose challenge, along with modest changes in insulin levels following TAK-875 treatment, pique interest in the mechanism of action for glucose lowering with this therapeutic. To assess the GSIS effects of GPR40 partial agonism and address how it may contribute to improved glycemia, we tested the effects of MK-2305, a potent and selective GPR40 partial agonist, on insulin secretion in the Goto Kakizaki (GK) rat. The GK rat is a polygenic model of type 2 diabetes where β-cell secretory defects result in reduced insulin secretion, impaired GSIS, hepatic insulin resistance, and hyperglycemia in the absence of obesity [11,12]. These features make the GK rat an ideal model to test the role of GSIS in GPR40 partial agonist efficacy. Once these GSIS effects were confirmed, we tested the effects of GPR40 agonist treatment on endogenous glucose production (EGP) and glucose uptake and metabolism into skeletal muscle, in order to further probe the mechanism by which GPR40 partial agonists improves glucose homeostasis.

## Materials and methods

### Compounds and reagents

MK-2305, a GPR40 partial agonist described previously [13], was used for *in vivo* evaluations. Rosiglitazone (catalogue # 71740) was purchased from Cayman Chemicals (Ann Arbor, MI). Rodent Diet #5001 was purchased from LabDiets (Richmond, IN). Compound was formulated into Rodent Diet #5001 by Research Diets Inc. (New Brunswick, NJ).

### Animal use and care

Male GK rats at 8 weeks of age and age matched Wistar Kyoto (WKY) lean controls (Taconic Farms Inc., Germantown, NY) were housed in pairs on a 12 h light cycle (lights on 7 A.M.– 7 P.M.). Female rats or mice were not used in these studies to avoid the potential for confounding effects of asynchronous estrus cycling on metabolic measurements; thus conclusions drawn from results may only apply to males until females are tested in future studies. Rats were allowed access to rodent chow and water ad libitum and acclimated for at least 1 week prior to study. All procedures were approved by the Merck & Co., Inc., Kenilworth, NJ, USA, Institutional Animal Care and Use Committee.

### *In vitro* receptor potency assays

#### Functional studies

CHO Jump-IN cells stably expressing rat GPR40 or HEK293 Jump-IN cells stably expressing rat GPR120 were cultured in DMEM media supplemented with 10% dialyzed FBS, non-essential amino acids, penicillin/streptomycin, 10 μg/ml Blasticidin S HCL and 200 or 100 μg/ml Hygromycin B, respectively (Life Technologies). Cell stocks were maintained and grown in a sub-confluent state using standard cell culture procedures. The day before the experiment, the cells were harvested with non-enzymatic cell dissociation buffer and re-suspended in DMEM supplemented as described above at 0.15 million cells per ml. A sterile PerkinElmer Culturplate-384 or a 384-well Corning BioCoat Poly-D-Lysine coated microplate, respectively, was then seeded with 7,500 cells in a volume of 50 μl per well. The seeded plates were incubated overnight at 37°C in a humidified environment.

On the day of the experiment, the growth media was removed from the assay plate by centrifugation using a BlueCatBio BlueWasher, protocol #21 “Light Dry” and 10 μl of IP1 stimulation buffer (prepared according to the Cisbio IP-one Tb HTRF kit) is added to each well. Test compounds were dissolved and serially diluted in DMSO as 200X the final assay concentration and 50 nl of the compound dilution was acoustically added to each well of the assay plates. The plates were then incubated for 60 minutes at room temperature and 10 μl of detection buffer (prepared according to the IP-one Tb HTRF kit) is added to each well of the assay plate. The plates were then incubated for one additional hour at room temperature. After the final incubation, the plates were read in a Perkin Elmer Envision with a method designed for HTRF assays (320 nm excitation, dual emission 615 and 655nm). For each assay, a standard curve plate in which IP1 is titrated is also included. All fluorescent readings (using the 655/615nm ratio) are back calculated to a concentration of IP1 using the IP1 standard curve and the percent activity at each concentration of test compound is determined using 0% activation (basal activity) determined in those wells that contain DMSO alone, while 100% activity is determined in wells that contained a concentration of agonist know to maximally activate GPR40 or GPR120. The % activation is then plotted versus the concentration of test compound and the dose response curve fitted to a standard 4-paramater non-linear regression model using a custom in-house developed software package. Maximal % activation and EC_50_ are then determined for each test compound.

CHO cells stably expressing human GPR41 or human GPR43 were harvested with non-enzymatic cell dissociation buffer and re-suspended in LANCE stimulation buffer (prepared as described by PE) at 0.25 million cells per ml (3.3 million cells per ml for GPR43) and 6 μl was then plated into each well of a 384-well Culturplate. Compounds were serially diluted in DMSO and then diluted further into stimulation buffer containing 2 μM forskolin (Sigma) at 2-times final concentration in the assay. 6 μl of the diluted compounds were then added to the assay plate and the plate incubated for 60 mins at room temperature. After the incubation, 12 μl of detection reagent (prepared as described by PE) was added to each well and the plates incubated one additional hour at room temperature. After the final incubation, the plates were read in a Perkin Elmer Envision reader with a method designed for Time-resolved fluorescence (TRF) assays (340 nm excitation, dual emission 615 and 655 nm). For each assay, a cAMP standard curve plate was also included. All fluorescent readings (using the 655/615 nm ratio) were converted to cAMP concentration using the cAMP standard curve and the percent activity at each concentration of test compound was determined using 0% activation (basal activity) determined in those wells containing DMSO alone, while 100% activity was determined in wells containing a concentration of agonist know to maximally activate the receptor.

Rat PPARα, PPARδ, and PPARγ activities were determined by Indigo Biosciences (State College, PA) cell-based nuclear receptor profiling service.

### *In vitro* receptor glucose stimulated insulin secretion (GSIS) assay

Pancreatic islets of Langerhans were isolated from wild-type and GPR40−/− mice (littermates) by collagenase digestion and discontinuous Ficoll gradient separation [14]. The islets were cultured overnight in RPMI-1640 medium with 11 mmol/l glucose to facilitate recovery from the isolation process. Insulin secretion was determined by a 1 hr static incubation in Krebs-Ringer bicarbonate (KRB) buffer in a 96-well format as previously described [5,15]. Briefly, islets were first preincubated in KRB medium with 2 mmol/l glucose for 30 min and were then transferred to a 96-well plate (three islets/well) and incubated with 200 μl of the KRB medium with 2 or 15 mmol/l glucose in the presence or absence of testing compounds for 60 min. The buffer was removed from the wells at the end of the incubation and assayed for insulin levels using the Ultrasensitive Rat Insulin ELISA kit (ALPCO, Salem, NH). All insulin secretion data were normalized by total islet numbers (ex: three islets/well), so the unit is secreted insulin (ng/ml) per islet.

### GK rat acute oral glucose tolerance test (OGTT)

On the day of the study, male GK rats were bled via tail snip, and blood glucose was measured via glucometer (OneTouch Ultra, Lifescan Inc., Milpitas, CA). Fed glucose was measured at 8 A.M. (lights on 7 A.M.– 7 P.M.) and used to select and sort animals into treatment groups (n = 8/ GK group, plus n = 6/WKY control group). Animals were then placed into clean cages, and food was removed. After a 5 hr fast, glucose was measured at time = -60 min, followed by administration of vehicle plus or minus compound via oral gavage (PO) at a dosing volume of 5 ml/kg. Dosages from 0.3–10 mg MK-2305 / kg body weight were tested. Both the GK and WKY control groups received vehicle (0.5% methylcellulose). Blood glucose was measured again (time = 0) immediately prior to a dextrose challenge of 1 g/kg at 10 ml/kg dose volume (Dextrose, Hospira Inc., Lake Forrest IL) and then again at 20, 40, 60, and 120 min after the glucose challenge. Insulin was co-measured to test the glucose-dependent secretory response to MK-2305 delivered acutely PO. To capture insulin secretion during the OGTT, blood was collected at t = -60, 0, 20, 40, 60 min post glucose challenge. The blood glucose excursion profile from t = 0 to t = 120 min was used to integrate an area under the curve (AUC) for each treatment. The plasma insulin excursion profile from t = 0 to t = 60 min was used to integrate an AUC for each treatment. Values of percent inhibition vs. vehicle for each treatment were generated from the AUC data. Maximally efficacious dose (MED) was defined as the minimum dosage generating a maximum inhibition of glucose excursion achieved with this compound in the OGTT assay.

### GK rat chronic efficacy

At the start of the study, fed glucose was measured at 8 A.M., and animals were selected and sorted into treatment groups based on ambient glucose (n = 10/male GK group, n = 6/male WKY control group). Animals were then placed on diet (LabDiet # 5001) that contained, or was devoid of, compound at the daily doses indicated. Fed blood glucose, food intake, and body weights were determined on days 0, 1, 3, 7, and 14 of study at 8 A.M. On days 7 and 14, food was removed for 5 h and plasma glucose was measured. Percent inhibitions were calculated as the percent reduction of blood glucose from the average value for vehicle controls. On day 14, blood was collected from the tail vein (20 μl) after the morning glucose measurement and after 5 h fast from a subset of n = 3 rats per treatment group to determine the ambient and fasted exposure of the compounds in circulation. The blood was stored at 4°C in 0.1 M sodium monocitrate for subsequent analysis. Animals were then re-fed their respective diet treatments. On day 20 of treatment at 8 A.M., blood glucose was measured and then animals were euthanized via CO_2_ asphyxiation. Blood and liver tissue were sampled, processed, and stored at -80°C until analysis.

In a parallel set of animals (n = 30) treated with standard diet or MK-2305 at 30 mg/kg diet, an OGTT with insulin co-measurement was performed on Day 13. On the day of OGTT, n = 10 vehicle animals and n = 10 MK-2305-treated animals were administered 0.5% methylcellulose PO at a dosing volume of 5 ml/kg. For an acute comparison, n = 10 vehicle animals were administered MK-2305 at 30 mg/kg PO. The procedure for an acute OGTT was identical to that described above.

### Isolated perfused liver studies

To evaluate whether GPR40 partial agonist treatment produced a direct effect on hepatic glucose production, we performed isolated perfused liver studies with ^13^C NMR spectroscopy in which GPR40 agonists were dosed directly to the liver *ex vivo*. Mouse livers were chosen instead of rat livers due to size restrictions of the perfused liver apparatus. The db/db mouse model was used in these studies as they have been shown to be a model of hepatic glucose overproduction [16], similar to the GK rat [17]. A detailed description of the perfused liver technique can be found elsewhere [18]. Briefly, db/db mice were anesthetized (Nembutal IP, 100 mg/kg), the portal vein was cannulated and tied off, and the liver was excised and perfused with a pre-oxygenated Krebs-bicarbonate buffered solution. The liver was then placed into a 20 mm NMR tube and the gluconeogenic substrate [2-^13^C] pyruvate (10 mM) was added to the perfusate plus either MK-2305 (10 μM) or vehicle (0.1% DMSO). Serial ^13^C NMR spectra were then acquired (30° pulse, D1 = 560 ms, NS = 800 averages, broadband GARP ^1^H decoupling, 11 min/spectrum) for 66 minutes, during which time the conversion of [2-^13^C] pyruvate (206 ppm) to [1-^13^C] glucose (96.8 ppm), [1-^13^C] glycogen (100.6 ppm), and [2-^13^C] lactate (69.4 ppm) was measured in real time via an increase of the ^13^C NMR resonances for each product. These NMR signals were then converted to absolute units (μmoles) by comparison to ^13^C NMR spectra of standard solutions acquired under identical conditions. Additional correction factors were included for ^13^C-glycogen and ^13^C-glucose to account for the fact that position 2 in pyruvate can end up at position 1, 2, 5, or 6 in glucose/glycogen due to the fumarase and triose phosphate isomerase reactions. The slope of each signal versus time was then used to calculate the following fluxes: glucose production, glycogen synthesis, and lactate production.

### Endogenous glucose production *in vivo*

To evaluate whether MK-2305 treatment produced an effect on EGP in vivo, a ^2^H/^13^C dual tracer approach was used to calculate EGP and its contributing sources. On the day of the study, GK rats were bled via tail snip, and blood glucose was measured via glucometer. Ambient glucose was measured at 8 A.M. and used to sort animals into treatment groups (n = 8 per group). 0.9% NaCl salinated D_2_O (Sigma-Aldrich, St. Louis, MO) was administered to all animals via IP injection at dose volume of 20 ml/kg. Animals were then placed in clean cages and food was removed. After a 5 hour fast, blood glucose was measured and animals were dosed PO with MK-2305 at 10 mg/kg (5 ml/kg) or vehicle (0.5% methylcellulose) immediately followed by 50 mg/kg [U-^13^C] glucose (Sigma-Aldrich) IP at 5 ml/kg. Blood was collected via tail snip at time points from 60–150 min post treatment to measure blood glucose as well as endogenous (^12^C-glucose) and exogenous (^13^C-glucose) via GCMS. Following the last tail blood collection, rats were anesthetized with isoflurane and cardiac blood was collected into K_2_EDTA tubes (Becton, Dickinson and Co., Franklin Lakes, NJ).

The serially collected blood samples were analyzed for ^13^C-glucose enrichment using GC-MS analysis. The % ^13^C glucose enrichments were then combined with the total glucose readings to calculate the concentration of ^13^C-glucose, and these values were modeled with a two-compartment model of whole body glucose metabolism to calculate the average EGP for the period from 0–150 minutes [19].

Terminal blood samples were analyzed for relative ^2^H enrichment in plasma glucose using the monoacetone glucose method [20]. Briefly, samples were extracted with 70% perchloric acid and centrifuged at 25,000 rcf for 10 min at 4°C. The supernatant was collected and the pH was adjusted to 6–8 using KOH. The samples were then lyophilized overnight. To convert plasma glucose to monoacetone glucose (MAG), acetone and concentrated sulfuric acid were added to the lyophilized samples and stirred for 4 hours at room temperature. Water was then added and the pH adjusted to 2.0 with 3M Na_2_CO_3_. Samples were stirred moderately for 18 hours at room temperature, the pH was adjusted to 7 with 3M Na_2_CO_3_, and the samples were completely dried by speed vacuum concentration. The resulting MAG samples were then extracted in hot ethyl acetate, dried overnight under a stream of N_2_ gas, purified using Discovery DSC-18 SPE columns (6 ml, 1 g bed weight), and freeze dried. Purified MAG samples were solubilized in 180 μl 90% acetonitrile for ^2^H NMR analysis which was performed using the lock channel of a 950 MHz Bruker NMR spectrometer (David H Murdock Research Institute, Kannapolis, NC). Acquisition parameters were as follows: 90° pulse, D1 = 1 s, ^1^H broadband decoupling, NS = 3600 averages, acquisition time ~ 1 hr. The ^2^H NMR spectra of the MAG samples were then analyzed using in–house developed Matlab (The Mathworks Inc, Natick, MA) to model the NMR peaks with a Lorentzian line shape, and the results were used to calculate integrals for each of the seven NMR resonances in the MAG spectrum. Peaks 2, 5, and 6s were then used to calculate the relative sources of EGP (i.e. gluconeogenesis from TCA cycle substrates, gluconeogenesis from triose phosphates, and glycogenolysis) and these values were combined with the total EGP measurement to calculate absolute fluxes of glucose production.

### ^13^C-labeled OGTT

GK rat OGTT procedures were followed as above. Animals were treated PO at 5 ml/kg with MK-2305 at 10 mg/kg or vehicle at time = -60 min. At time = 0, animals were challenged PO at 10 ml/kg dose volume with [1-^13^C] glucose prepared in distilled water at 1 g/kg (Sigma-Aldrich). Blood glucose was measured pre-treatment and at t = 0, 20, 40, 60, 120 minutes post challenge. Following the blood glucose measurement at time = 120 min, animals were anesthetized with Isoflurane (Isothesia, Henry Schein, Dublin, OH) and samples of liver and skeletal muscle were collected. Tissue samples were processed using a methanol/water based extraction procedure as follows. Frozen tissue was weighed and added to tubes containing 80% methanol at a ratio of 100 mg tissue per mL 80% methanol. The samples were homogenized at 4°C using a Polytron homogenizer equipped with a 12 mm stainless steel head. Samples were then centrifuged at 25,000 rcf for 10 min at 4°C, the supernatant collected, and the pellet extracted again using an equivalent volume of 80% methanol. The supernatants from both extractions were pooled and the methanol evaporated under nitrogen. The samples were then reconstituted in 180 μl 0.1M sodium phosphate in D_2_O (pH 7.4) for NMR analysis. ^13^C NMR of tissue extracts was performed on a 600 MHz Bruker NMR spectrometer (David H. Murdock Research Institute) using the following parameters: 45° pulse, D1 = 0.2 sec, NS = 3200 averages, ^1^H broadband decoupling (WALTZ-16), acquisition time ~45 min. The following ^13^C NMR signals were recorded: glucose-6-phosphate C1 (97 ppm), lactate C3 (21 ppm), alanine C3 (17.1 ppm). These NMR signals were integrated and converted to absolute units (μmoles) using ^13^C NMR spectra from standard solutions of each metabolite of known concentration acquired under identical conditions. Glycogen is a primary site of glucose disposal in both liver and muscle, however glycogen was not preserved in our tissue extraction protocol. To quantify tissue ^13^C-glycogen, a separate piece of tissue from each sample was analyzed. Briefly, tissue samples were saponified in hot KOH, and this was followed by glycogen precipitation with NaSO_4_ and ethanol, and then degradation with amyloglucosidase. The resulting glycogen-derived glucose solution was analyzed for total glucose using a glucose oxidase / horseradish peroxidase assay kit and fluorescence-based product detection according to the manufacturers’ instructions (BioVision Glycogen Assay Kit, Milpitas, CA). Fractional ^13^C enrichment in glucose was performed using GC-MS and this data was combined with the total glucose measurements to yield the tissue concentration of ^13^C-glycogen.

### Measurement of plasma and tissue metabolites

Plasma was analyzed for triglycerides, NEFA, and HbA1C using a P Modular Roche Clinical Chemistry analyzer. Liver triglycerides were extracted as described by Bligh and Dyer [21], and were measured using the Infinity triglyceride assay kit (Thermo Scientific, Rockford, IL). Plasma insulin levels were measured via EIA (Mesoscale Discovery ELISA Kit, Gaithersburg MD).

### Data analysis

All data are presented as mean ± SEM. Comparisons among groups were made using one-way ANOVA followed by post hoc analysis of multiple comparisons or when comparing only two groups, treated vs. vehicle were compared via by unpaired Student's *t* test. *P* < 0.05 was regarded as statistically significant.

## Results

### MK-2305 is a potent, selective GPR40 partial agonist that stimulates GSIS

**Fig 1:**
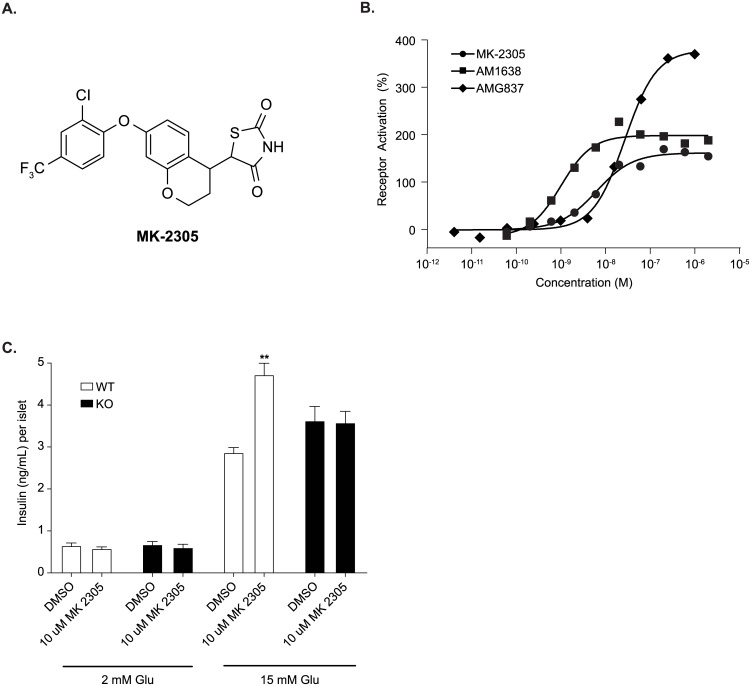
In vitro and ex-vivo pharmacology of MK-2305. (A) Structure of the synthetic GPR40 partial agonist MK-2305. (B) Dose-response curves for MK-2305 were generated monitoring IP accumulation in CHO cells expressing rat GPR40. Data are expressed as a percentage of the control response of an in-house partial agonist, and fitted to a standard 4-parameter non-linear regression model. EC_50_’s were determined for each test compound using a custom in-house developed software package. Each experiment was multiple times with a representative graph shown. The mean parameters of these and other individual experiments are shown in Table 1. (C) Effect of MK-2305 on GSIS in isolated GPR40 WT and KO islets under high (15 mM) and not basal (2 mM) glucose. Data provided are means +/- SEM. Data were analyzed via ANOVA followed by Bonferroni multiple comparisons test. **p<0.01compared to DMSO treated islets under 15 mM glucose.

A synthetic GPR40 partial agonist (MK-2305, Fig 1A) was developed possessing nanomolar potency (6 ± 2.9 nM, 166% activation, *N* = 29), as determined in an IP1 functional assay performed in a CHO cell line stably expressing the rat GPR40 receptor (Fig 1B, Table 1). MK-2305 is a partial agonist of the GPR40 receptor given that the degree of activation is similar to other described GPR40 partial agonists such as AMG837 [22] and significantly less than that observed for full agonists such as AM1638 (Fig 1B). Binding assays indicate that MK-2305 binds to the same site as other GPR40 partial agonists such as AMG837 and TAK-875 [23] and augments the binding of AgoPAM’s such as AM1638 [13]. MK-2305 had a free fraction of 2.6% measured in a protein binding assay in mouse plasma. MK-2305 was highly selective for activation of the rat GPR40 receptor (FFA1) vs. other receptors known to improve glucose metabolism such as GPR120 (FFA4), GPR41 (FFA3), GPR43 (FFA2), PPARα, PPARδ, PPARγ (Table 1).

**Table 1:** *In vitro* pharmacology of MK-2305.

Receptor	EC_50_ (nM)	% Activation
Rat GPR40 (FFAR1)	6 ± 2.9	166 ± 31^1^
Rat GPR120 (FFAR4)	>10, 000	9 ± 7 @ 10 μM
Human GPR41 (FFAR3)	12711	85 @ 25 μM
Human GPR43 (FFAR2)	>25, 000	61 @ 25 μM
Rat PPARα	>10, 000	-0.2 @ 10 μM
Rat PPARδ	>10, 000	-0.1 @ 10 μM
Rat PPARγ	>10, 000	3.1 @ 10 μM

MK-2305 potency and selectivity is examined against the GPR40 receptor, other FFAR family members, and peroxisome proliferator-activated receptors (PPAR). All receptors are rat orthologs, except GPR41 and GPR43 which are human. Values are mean ± SD.

^1^Relative to AMG837 (% Activation, 173%) and AM1638 (%Activation, Act 373%).

In isolated islet studies, MK-2305 was observed to acutely stimulate GSIS in WT islets, but not in GPR40 KO islets under high (15 mM) but not basal (2 mM) glucose (Fig 1C). This demonstrates that GPR40 is required to mediate the GSIS effects of MK-2305.

### GPR40 partial agonist acutely improves glucose tolerance and GSIS

**Fig 2:**
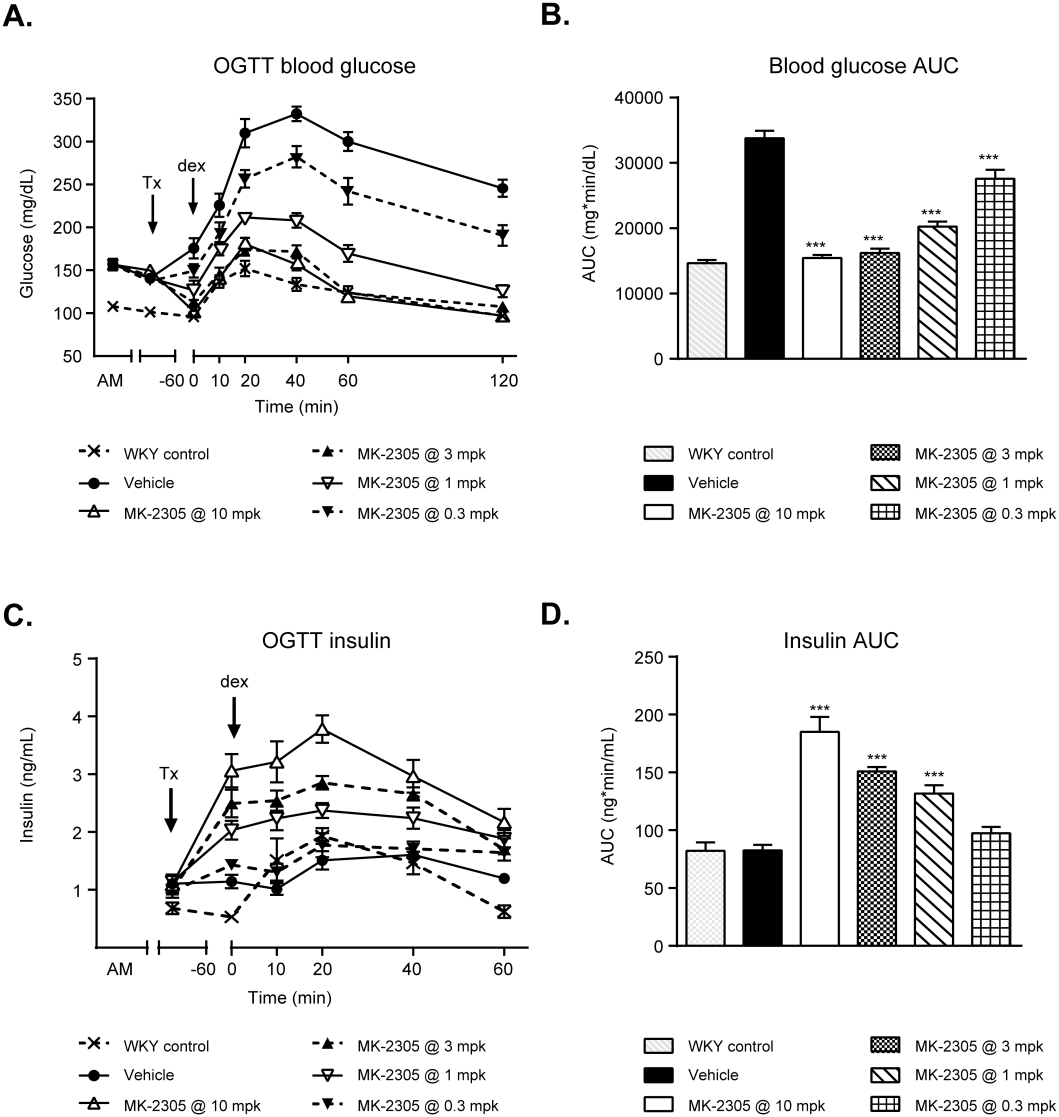
Effects of MK-2305 on oral glucose tolerance in GK rats. (A) Blood glucose time course in GK rats treated acutely with MK-2305, followed by an OGTT. (B) AUC data for glucose vs. time post-challenge data in (A). (C) Plasma insulin vs. time data for the OGTT shown. (D) Plasma insulin AUC data for the OGTT. Statistical comparisons of AUC for glucose or insulin across treatment groups were compared via ANOVA followed by Dunnetts post hoc analysis where MK-2305 treated groups were compared to vehicle. ***p<0.001.

GK rats treated acutely with MK-2305 demonstrated a dose dependent reduction in plasma glucose during an OGTT (Fig 2A and 2B). MK-2305 significantly decreased AUC at doses between 10–0.3 mg/kg (by 54% to 18%, respectively, all *p <* 0.001 vs. vehicle). The MED for MK-2305 was 3 mg/kg, which elicited a 40% reduction in AUC from vehicle (p < 0.001). To determine whether these improvements were associated with an increase in glucose stimulated insulin secretion (GSIS), plasma insulin was co-measured during the OGTT. GK rats treated with MK-2305 showed significant dose dependent increases in plasma insulin compared to vehicle controls during the OGTT at doses of 10, 3, and 1 mg/kg (Fig 2C and 2D; 124%, 83%, and 59% increases, respectively, all *p <* 0.001).

### GPR40 agonist lowers fasting and fed glucose following chronic administration

**Fig 3:**
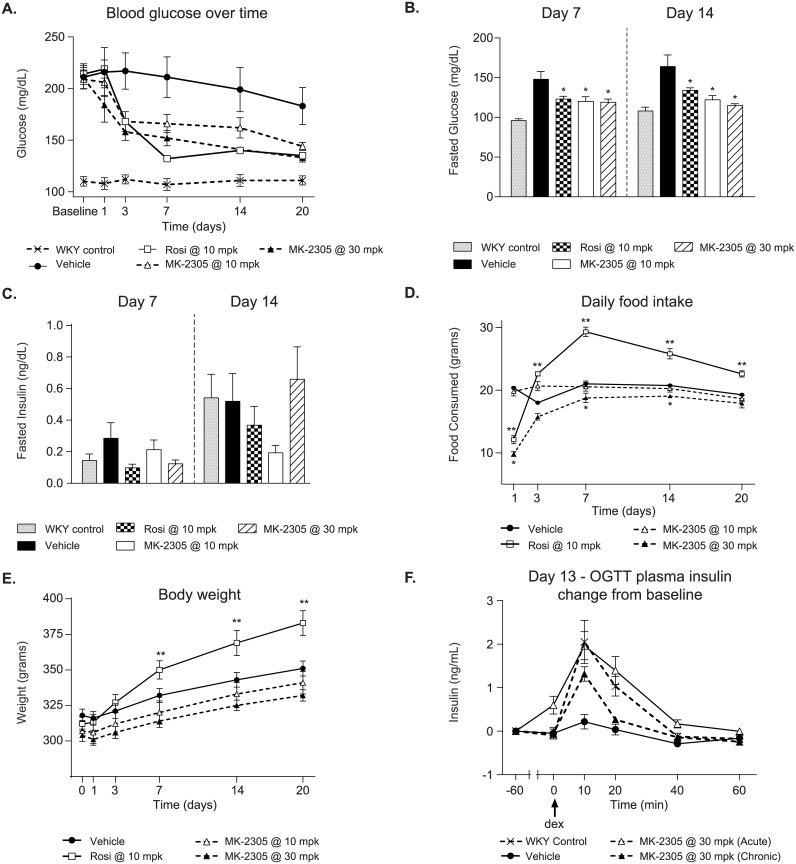
Effects of chronic treatment with MK-2305 in the GK rat. (A) Morning blood glucose levels in GK rats treated with vehicle, 10, or 30 mg/kg of MK-2305, or 10 mg/kg rosiglitazone for 20 days in feed. (B) Fasted blood glucose levels on days 7 and 14 of the study were significantly reduced with MK-2305 and rosiglitazone treatment compared to vehicle controls. (C) Fasted plasma insulin levels on days 7 and 14. (D) Effects on food intake and (E) body weight during the chronic study. (F) Plasma insulin levels during a OGTT in chronically treated rats on day 13. Changes in blood glucose, food intake or body weight over time with MK-2305 or rosiglitazone vs. vehicle were analyzed by two-way ANOVA with repeated measures followed by Tukeys post hoc analysis. Changes in fasted glucose or insulin of glucose AUC were analyzed by one way ANOVA comparing MK-2305 or rosiglitazone treatments with vehicle followed by Dunnetts post hoc analysis. *p<0.05, **p<0.01.

To examine the chronic effects of a GPR40 partial agonist on glucose homeostasis, GK rats were treated with MK-2305 or the PPARγ agonist rosiglitazone for 20 days as an ad-mix in feed (Fig 3). On day 3, MK-2305 treated rats had a significant decrease in fed glucose (measured 1 hr post lights on) of 27% and 22% at 30 mg/kg and 10 mg/kg, respectively (*p < 0*.*05*). Similar effects on glycemia were observed with rosiglitazone at 10 mg/kg (22% vs. vehicle, *P < 0*.*05*, Fig 3A). The effects of both treatments on fed glucose were maintained through day 20.

In addition to fed glucose measures, blood glucose and plasma insulin were also measured after a 5 hr fast (Fig 3B and 3C). Compared to vehicle, GK rats treated with 30 or 10 mg/kg MK-2305 exhibited significant reductions in fasting blood glucose of 19% for both doses on day 7 and 30% and 26%, respectively, on day 14 (Fig 3B, *p* < *0*.*05*). Rats treated with rosiglitazone demonstrated a similar reduction in blood glucose such that 10 mg/kg rosiglitazone treatment decreased blood glucose by 16% on day 7 and 19% on day 14 (*p* < *0*.*05*). Interestingly, there were no significant effects of either MK-2305 or rosiglitazone on plasma insulin collected at these time points (Fig 3C). However, in the parallel set of animals, MK-2305 increased plasma insulin compared to vehicle both acutely and chronically after a glucose challenge (Fig 3F).

MK-2305 plasma exposures for the 10 and 30 mg/kg doses were ~15 μM and ~39 μM, respectively, and were similar at both the fed and fasted time points. Rosiglitazone exposures were ~3 and 0.7 μM for the fed and fasted time points, respectively for rats treated with the 10 mg/kg dose.

Changes in food intake and body weight were assessed throughout the course of the chronic study (Fig 3D and 3E). While treatment with MK-2305 lowered glucose at both 10 and 30 mg/kg, the 10 mg/kg dose had no significant effect on food intake or body weight, whereas the 30 mg/kg dose resulted in a reduction in food intake that persisted through day 14 of study (Fig 3D, *p* < *0*.*05*). The effect of 30 mg/kg MK-2305 on food intake did not correspond to changes in body weight relative to vehicle controls and lost statistical significance on day 20 of study (Fig 3E). In contrast, rats treated with rosiglitazone demonstrated a reduction in food intake on day 1 of study that changed to a significant and consistent increase in food consumption beginning on day 3 (Fig 3D, *p* < *0*.*01*). Food intake changes observed with rosiglitazone treatment were accompanied by a steady increase in body weight that reached statistical significance on days 7–14 of study (Fig 3E, *p* < *0*.*01*).

**Fig 4:**
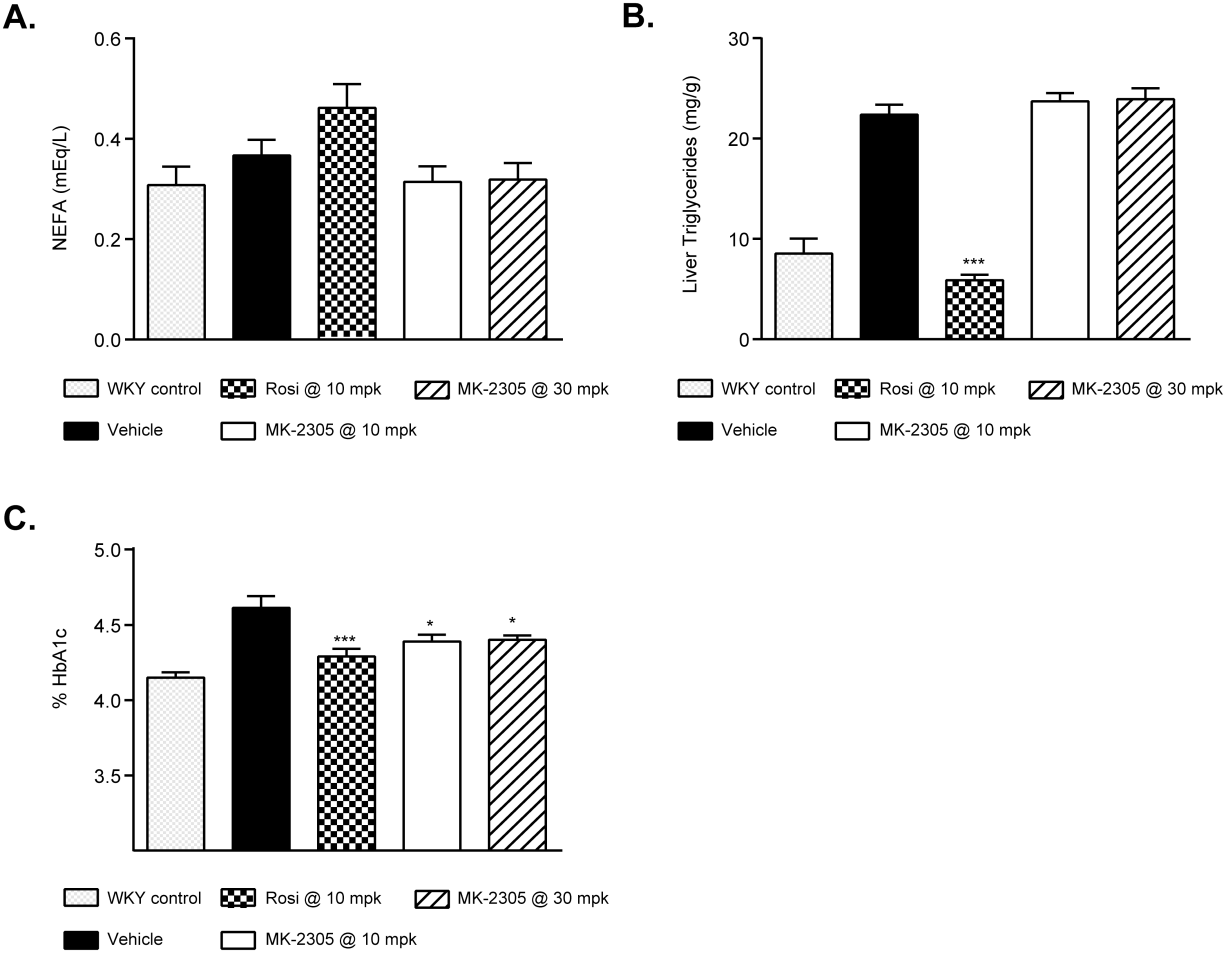
Effects of chronic treatment with MK-2305 on lipids and HBA1c in the GK rat. Effects of vehicle, Rosiglitazone, and MK-2305 on rat (A) NEFA, (B) liver TG, and (C) HbA1c following 20 days of treatment. Data were analyzed via ANOVA followed by Dunnets test comparing Rosiglitazone or MK-2305 treatments with vehicle. *p<0.05, ***p<0.001.

On study day 20, rats were euthanized to collect blood and tissues for analysis of plasma metabolites (Fig 4). MK-2305 treatment had no effect on plasma non-esterified fatty acid (NEFA) levels at either dose. Rosiglitazone treatment increased NEFA from 0.37 ± 0.03 to 0.48 ± 0.05 mEq/L but was not significant vs. vehicle treated GK rats (Fig 4A). MK-2305 had no effect on liver triglyceride levels, while rosiglitazone showed a significant decrease compared to vehicle controls (*p < 0*.*001*, Fig 4B). In line with the effects of the MK-2305 and rosiglitazone on glucose parameters (Fig 3A and 3B), circulating Hb_A1C_ levels were significantly reduced in all treatment groups (MK-2305 *p < 0*.*05*, rosiglitazone *p < 0*.*001*, Fig 4C).

### Isolated perfused liver studies

**Fig 5:**
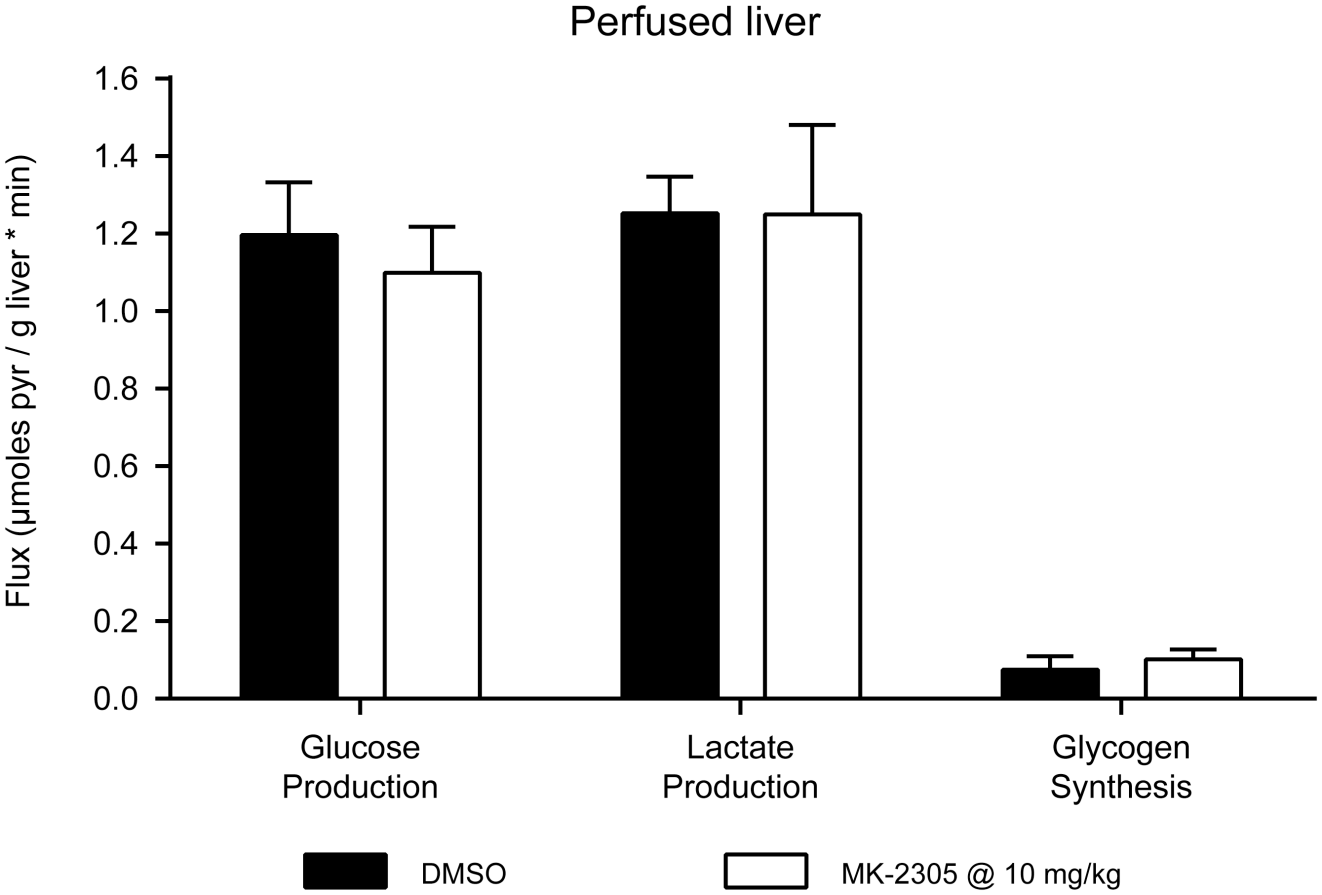
Effects of MK-2305 on glucose metabolism in perfused mouse livers. Effect of 10 μM MK-2305 or DMSO on the conversion of [2-^13^C] pyruvate to ^13^C-glucose, ^13^C-glycogen, and ^13^C-lactate in perfused db/db mouse livers. MK-2305 treatments were compared to vehicle for each endpoint via students ttest.

To investigate the direct effects of MK-2305 on hepatic gluconeogenesis, we performed isolated perfused liver studies using the gluconeogenic substrate [2-^13^C] pyruvate. Acute treatment of the liver with MK-2305 at 10 μM (Fig 5) produced no effects on the conversion of [2-^13^C] pyruvate to ^13^C-glucose, ^13^C-glycogen, or ^13^C-lactate, suggesting that the GPR40 partial agonist MK-2305 had no *direct* effect on hepatic gluconeogenesis.

### Endogenous glucose production in vivo

**Fig 6:**
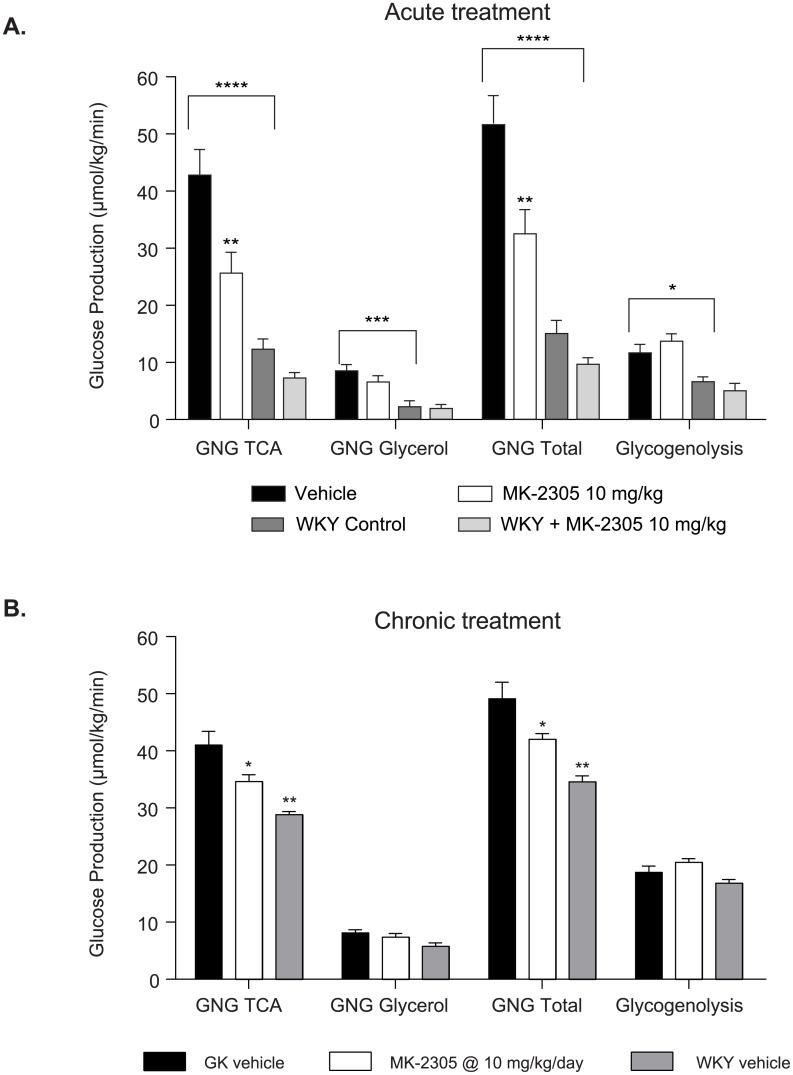
Effect of (A) acute treatment and (B) chronic treatment with 10 mg/kg MK-2305 or vehicle on endogenous glucose production (EGP) from various substrates in the GK or WKY rats. Data were analyzed via ANOVA followed by Tukey’s multiple comparisons test comparing MK-2305 treatment to vehicle within GK or WKY rats or comparing WKY rats treated with vehicle to GK rats treated with vehicle. *p< 0.05, **p<0.01, ***p<0.005, ****p<0.001.

To investigate the acute and chronic mechanisms of MK-2305-induced glucose lowering, we performed dual tracer studies using D_2_O and ^13^C-glucose to measure EGP *in vivo* (Fig 6). Acutely, MK-2305 significantly reduced total EGP (32.9 ± 3.95 vs 52.0 ± 4.82 μmol/kg/min, *p < 0*.*05*), EGP from gluconeogenic substrates entering at the TCA cycle (26.0 ± 3.36 vs. 43.2 ± 4.17 μmol/kg/min, *p < 0*.*01*), and EGP from gluconeogenic substrates entering as triose phosphates (6.90 ± 0.80 vs. 8.82 ± 0.81 μmol/kg/min, *p < 0*.*05*) (Fig 6A). MK-2305 did not acutely affect EGP derived from glycogenolysis. Taken together, these data indicate that GPR40 agonist acutely reduces EGP *in vivo*, primarily through reductions in gluconeongenesis. Similar results were seen after chronic administration of MK-2305 (Fig 6B). Also noteworthy is the increased EGP from gluconeogenic substrates in the diabetic GK rats compared to WKY control rats. Acute effects of MK-2305 were also examined in WKY rats. Unlike the in the diabetic GK rat, the effects of MK-2305 on EGP in the WKY were subdued and though trending in the same direction were not statistically significant. Due to the narrow window for effect size we opted not to include treated WKY rats in the chronic study.

### Glucose uptake in liver and muscle using ^13^C-GTT

**Fig 7:**
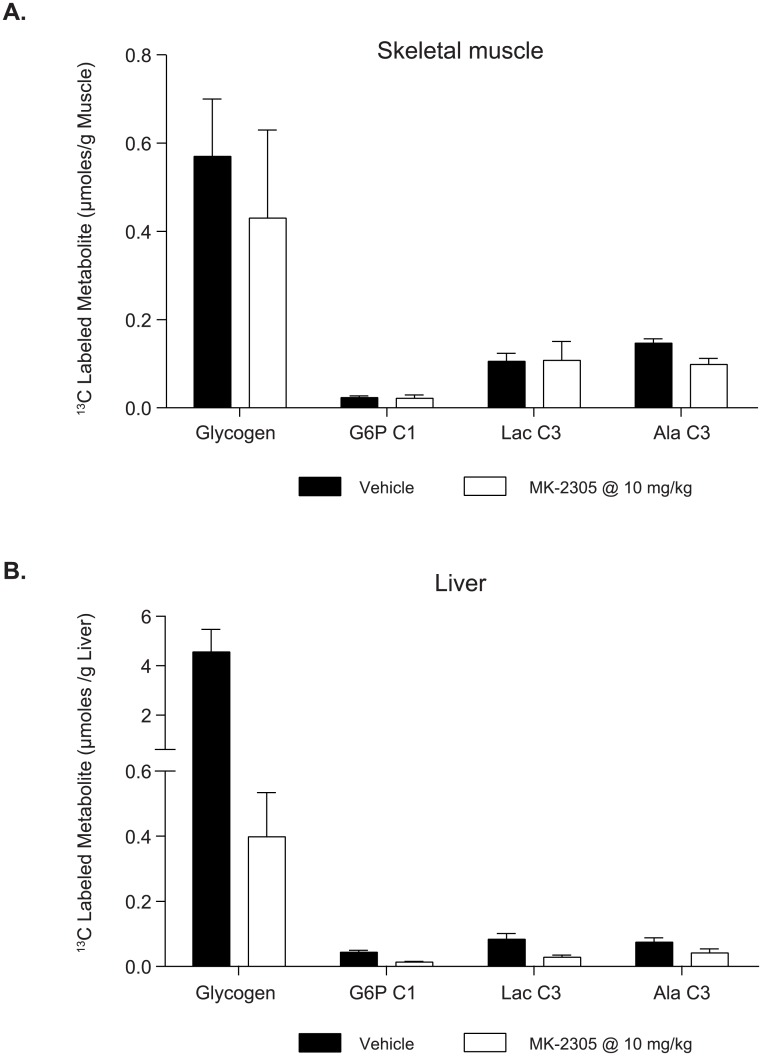
Uptake and metabolic conversion of [1-^13^C] glucose in liver (A) and muscle (B) following acute treatment with MK-2305 or vehicle. Data were analyzed via students ttest comparing MK-2305 treatment vs. vehicle for each metabolite in each tissue measured.

To investigate the effects of MK-2305 on glucose uptake following a GTT, we performed [1-^13^C] glucose OGTT studies and measured the resulting distribution of ^13^C-labeled metabolites (i.e. those derived from the exogenous [1-^13^C] glucose) in muscle and liver. In muscle (Fig 7A), MK-2305 treatment produced no significant effects on the disposal of ^13^C-glucose into the metabolites shown. In liver (Fig 7B), GPR40 treatment produced significant decreases in the disposal of ^13^C-glucose into ^13^C-G6P, ^13^C-glycogen and the glycolytic products ^13^C-lactate and ^13^C-alanine. While we acknowledge that other ^13^C labeled metabolites were present in the ^13^C NMR spectra, all were of lower abundance, and none showed different trends form the ones reported here. Taken together, these data indicate that the improvements in the GTT response observed with acute GPR40 partial agonist treatment are likely not mediated by enhanced glucose uptake in either muscle or liver.

## Discussion

GPR40 activation with partial agonist MK-2305 leads to sustained reductions in hyperglycemia in the diabetic GK rat model accompanied by increases in glucose stimulated insulin secretion. Glucose lowering was maintained in the absence of changes in fasted insulin, food intake, or body weight. These data in rodents conform to the same basic trends observed when fasiglifam (TAK875), a GPR40 partial agonist, was administered to diabetic humans [9,10]. Interestingly, the large reductions in both fasting and non-fasting glucose levels in humans and rats treated with GPR40 agonists were accompanied by very modest increases insulin secretion that were observed in diabetics only under conditions of hyperglycemia (i.e. glucose challenge). Though modest, the ability to augment glucose stimulated insulin secretion was observed in both the GK rat and human diabetics. These similarities suggest that the GK rat may be a good model for exploring the underlying mechanisms by which GPR40 partial agonists mediate glucose lowering across species. In addition, the GK rat exhibits reduced glucose stimulated insulin secretion and increased EGP similar to the phenotypes observed in type 2 diabetic humans [12,17]. Taken together, these findings provide rationale for choosing the diabetic GK rat model to further our mechanistic insight into the glucose lowering effect of GPR40 partial agonists.

Treatment of GK rats with MK-2305 resulted in dose dependent reductions in blood glucose, which were coupled to decreased EGP. Elevations in EGP correlate with elevations in fasted glucose in humans and in rats and have been demonstrated to be an important contributor to fasting hyperglycemia [17,24]. As we did not see direct effects of MK-2305 agonists in the perfused liver, and GPR40 is not expressed in the liver [2] (and unpublished observations), we hypothesize that the changes in fasting plasma glucose in diabetics induced by GPR40 partial agonists may result from changes in liver metabolism secondary to changes in insulin secretion. Interestingly, GPR40 stimulated insulin secretion is observed only during hyperglycemia. Increases in insulin during a GTT are maintained following chronic treatment, demonstrating that the pancreas maintains enhanced GSIS responsiveness throughout GPR40 partial agonist treatment. Therefore we suspect that improved GSIS upon GPR40 partial agonist treatment may meter fasting as well as post prandial hyperglycemia in diabetics, leading to a reduction in EGP and normalization of circulating glucose. Since EGP is a major contributing factor to glycemia in both the glucose tolerance test and fasted state in the GK rat, as well as in diabetic humans, the link between GPR40 partial agonists, GSIS, EGP and reductions in glycemia provides a reasonable theory for how GPR40 partial agonists lower glucose [17,25]. Whether reductions in EGP accompany reductions in glycemia with GPR40 partial agonist treatment in diabetic humans remains to be tested.

The MK-2305-mediated reductions in EGP observed in GK rats were due primarily to decreases in hepatic gluconeogenic flux. This finding is of interest because most studies testing the effects of insulin on hepatic glucose metabolism have shown insulin decreases EGP through decreases in glycogenolysis. However, most of these studies have been conducted in lean healthy animals and humans [26–29]. It is unclear whether the effects of insulin on hepatic glucose metabolism in healthy subjects apply to those observed in diabetics. One study comparing the effects of insulin on sources of EGP in healthy and type 2 diabetic humans demonstrated that under euglycemic clamp conditions, increases in circulating insulin within a physiologic range resulted in a 20% suppression of gluconeogenesis and complete blockade of glycogenolysis [30]. However in this study, hyperinsulinemia was applied under euglycemic conditions, a situation unlike that observed with GPR40 agonist treatment where increases in insulin are dependent on elevations in circulating glucose. Though we used the same ^2^H tracer methodology in rats that was used in humans, the physiological context under which we tested the effects of GPR40 partial agonism (fasted rats) was different than that used to study effects of insulin in human diabetics (euglycemic hyperinsulinemic clamp), making the results difficult to compare.

Studies defining the components of EGP in type 2 diabetic humans suggest that hyperglycemia is associated with increases in EGP resulting from increases in glycogenolysis, gluconeogenic flux, or both [31–34]. We and others have shown that elevated EGP is primarily a result of increased gluconeogenic flux in the hyperglycemic GK rat [17]. Therefore, GPR40 partial agonist-mediated decreases in EGP through this pathway may drive glucose lowering in both diabetic rats and humans. Experimental medicine studies in humans incorporating tracer methods mirroring those described here in the GK rat are viable and would certainly test the translation of the mechanism mediating glucose lowering via GPR40 agonists to humans.

In order to investigate whether GPR40 partial agonist-mediated reductions in gluconeogenesis were due to direct effect on the liver, we performed isolated perfused liver studies in which MK-2305 was administered directly to the isolated liver. Consistent with the lack of GPR40 receptor expression in the liver, we observed no direct effect of MK-2305 on the conversion of [2-^13^C]-pyruvate to ^13^C-glucose, a biomarker of gluconeogenesis, in these studies. The perfused liver technique is useful in evaluating the direct effects of novel compounds and mechanisms on glucose production for the following reasons: (1) glucose production rates in the perfused liver are similar to those observed *in vivo* and (2) direct enzymatic inhibitors of gluconeogenesis (e.g. 3-MPA and CS917, inhibitors of phosphoenolpyruvate carboxykinase (*PEPCK)* and fructose-1,6-bisphosphatase (*F-1*,*6-BPase*), respectively) produce marked acute reductions of gluconeogenesis in the perfused liver (data not shown). Thus it is unlikely that the observed lack of direct effect of GPR40 on hepatic gluconeogenesis is due to an artifact of the method.

Using additional tracer methodology, we also examined the effects of MK-2305 treatment on the distribution of ^13^C-labeled metabolites (i.e. those derived from the exogenous [1-^13^C] glucose) in muscle and liver during a ^13^C-glucose GTT. We found that MK-2305 treatment produced no significant effects on the disposal of ^13^C-glucose into metabolites in skeletal muscle. Though insulin is known to affect glucose uptake in skeletal muscle, we hypothesize that we did not observe additional increases in glucose uptake with GPR40 partial agonist treatment due to the modest effects they have on circulating insulin levels in this setting.

Interestingly, GPR40 partial agonist treatment did not increase glucose uptake into the liver. In fact, GPR40 agonist treatment resulted in decreased ^13^C-glucose uptake and conversion to ^13^C-G6P and ^13^C-glycogen in the liver. One possible explanation for this finding is that the observed decrease in hepatic glucose uptake was due to the initial drop in fasting glucose (from hyperglycemia to euglycemia) prior to the challenge with the 13C-glucose. Since elevations in plasma glucose levels are the primary driver of hepatic glucose uptake [35], and plasma glucose in MK-2305 treated rats was significantly lower than baseline at the time of the glucose challenge this MK-2305 mediated reduction in glucose prior to the GTT likely led to decreased hepatic glucose uptake observed. These data indicate that the glucose lowering observed with acute GPR40 treatment during a GTT is not mediated by glucose uptake. Based on these findings we find it reasonable to believe that the glucose lowering effects observed with MK-2305 treatment in these studies are predominantly driven by the underlying decreases in EGP described above.

In addition to the direct effects of insulin on the liver, insulin may regulate hepatic glucose metabolism through additional indirect effects. These indirect effects may be due in part to insulin-mediated suppression of lipolysis resulting in decreased circulating free fatty acids (FFAs). Insulin-mediated decreases in plasma FFAs reduce the supply of both the energy producing substrate and signal in the gluconeogenic process [36]. In fasted GK rats treated with MK-2305, FFAs initially decrease in concordance with decreases in circulating glucose (unpublished observations). However, upon chronic GPR40 partial agonist treatment, no changes in circulating FFAs are observed in the face of reductions in gluconeogenic flux. These data suggest that direct effects of insulin on the liver may be the predominant mechanism mediating the chronic effect of GPR40 partial agonists in the GK rat. Unlike humans, where levels of circulating free fatty acids are quite variable, the GK rat does not demonstrate elevations in free fatty acids relative to non-diabetic WKY controls. It would therefore be of interest to determine the relative importance of the direct vs. indirect mechanisms with respect to the effects of GPR40 partial agonism on hepatic regulation of glucose metabolism in diabetic humans vs. the GK rat.

In summary, these data demonstrate that GPR40 partial agonist treatment results in decreases in glucose accompanied by GSIS in a diabetic rodent model that recapitulates many of the results that have been reported in human clinical trials. Detailed evaluation of the mechanism of action of GPR40 partial agonists in the GK rat model revealed that a reduction in EGP via decreased gluconeogenic flux underlies the observed reduction in hyperglycemia. Investigations of GPR40 partial agonists for benefits beyond glucose lowering will aid in our understanding of this novel class of anti-diabetic agents. Determination of the translatability of the mechanisms for glucose lowering induced by GPR40 partial agonists in the GK rat to that of human diabetics should be possible using similar experimental paradigms and techniques to those described here. This preclinical dataset provides novel insight into the metabolic effects of GPR40 partial agonists, a therapeutic target of interest given it has proven to produce efficacy when administered to human diabetics.

## Supporting information

S1 File Data for Table1: In vitro pharmacology. (XLSX)

S2 File Data for Figure1B: In vitro pharmacology of MK-2305. (XLSX)

S3 File Data for Figure1C: Effect of MK-2305 on GSIS in isolated GPR40 WT and KO islets under high (15 mM) and not basal (2 mM) glucose. (XLSX)

S4 File Data for Figure2A: Blood glucose time course in GK rats treated acutely with MK-2305, followed by an OGTT. (XLSX)

S5 File Data for Figure2B: AUC data for glucose vs. time post-challenge data in (A). (XLSX)

S6 File Data for Figure2C: Plasma insulin vs. time data for the OGTT shown. (XLSX)

S7 File Data for Figure2D: Plasma insulin AUC data for the OGTT. (XLSX)

S8 File Data for Figure3A: Morning blood glucose levels in GK rats treated with vehicle, 10, or 30 mg/kg of MK-2305, or 10 mg/kg rosiglitazone for 20 days in feed. (XLSX)

S9 File Data for Figure3B: Fasted blood glucose levels on days 7 and 14 of the study were significantly reduced with MK-2305 and rosiglitazone treatment compared to vehicle controls. (XLSX)

S10 File Data for Figure3C: Fasted plasma insulin levels on days 7 and 14. (XLSX)

S11 File Data for Figure3D: Effects on food intake S12 File. Effects on food intake.
(XLSX)

S12 File Data for Figure3E: Body weight during the chronic study. (XLSX)

S13 File Data for Figure3F: The change in plasma insulin levels from baseline during a OGTT in chronically treated rats on day 13. (XLSX)

S14 File Data for Figure4A: Effects of vehicle, rosiglitazone, and MK-2305 on NEFA following 20 days of treatment in the GK rat. (XLSX)

S15 File Data for Figure4B: Effects of vehicle, rosiglitazone, and MK-2305 on rat Liver TGs following 20 days of treatment in the GK rat. (XLSX)

S16 File Data for Figure4C: Effects of vehicle, rosiglitazone, and MK-2305 on rat 5HBA1c following 20 days of treatment in the GK rat. (XLSX)

S17 File Data for Figure5: Effect of 10 M MK-2305 or DMSO on the conversion of [2-13C] pyruvate to 13C-glucose, 13C-glycogen, and 13C-lactate in perfused db/db mouse livers. (XLSX)

S18 File Data for Figure6A: Effect of acute treatment with 10 mg/kg MK-2305 or vehicle on EGP from various substrates in the GK or WKY rats. (XLSX)

S19 File Data for Figure6B: Effect of chronic treatment with 10 mg/kg MK-2305 or vehicle on EGP from various substrates in the GK or WKY rats. (XLSX)

S20 File Data for Figure7A liver glucose uptake and incorporation into metabolites. Uptake and metabolic conversion of [1-13C] glucose in liver following acute treatment with MK-2305 or vehicle.
(XLSX)

S21 File Data for Figure7B skeletal muscle glucose uptake and incorporation into metabolites. Uptake and metabolic conversion of [1-13C] glucose in muscle following acute treatment with MK-2305 or vehicle.
(XLSX)
